# Diversity Generator Mechanisms Are Essential Components of Biological Systems: The Two Queen Hypothesis

**DOI:** 10.3389/fmicb.2018.00223

**Published:** 2018-02-13

**Authors:** Eric Muraille

**Affiliations:** Laboratoire de Parasitologie, Faculté de Médecine, Université Libre de Bruxelles, Brussels, Belgium

**Keywords:** evolution, adaptation, Cairnsian dynamic, diversity generator, red queen, white queen

## Abstract

Diversity is widely known to fuel adaptation and evolutionary processes and increase robustness at the population, species and ecosystem levels. The Neo-Darwinian paradigm proposes that the diversity of biological entities is the consequence of genetic changes arising spontaneously and randomly, without regard for their usefulness. However, a growing body of evidence demonstrates that the evolutionary process has shaped mechanisms, such as horizontal gene transfer mechanisms, meiosis and the adaptive immune system, which has resulted in the regulated generation of diversity among populations. Though their origins are unrelated, these diversity generator (DG) mechanisms share common functional properties. They (i) contribute to the great unpredictability of the composition and/or behavior of biological systems, (ii) favor robustness and collectivism among populations and (iii) operate mainly by manipulating the systems that control the interaction of living beings with their environment. The definition proposed here for DGs is based on these properties and can be used to identify them according to function. Interestingly, prokaryotic DGs appear to be mainly reactive, as they generate diversity in response to environmental stress. They are involved in the widely described Red Queen/arms race/Cairnsian dynamic. The emergence of multicellular organisms harboring K selection traits (longer reproductive life cycle and smaller population size) has led to the acquisition of a new class of DGs that act anticipatively to stress pressures and generate a distinct dynamic called the “White Queen” here. The existence of DGs leads to the view of evolution as a more “intelligent” and Lamarckian-like process. Their repeated selection during evolution could be a neglected example of convergent evolution and suggests that some parts of the evolutionary process are tightly constrained by ecological factors, such as the population size, the generation time and the intensity of selective pressure. The ubiquity of DGs also suggests that regulated auto-generation of diversity is a fundamental property of life.

## The Fundamental Question of How and Why Diversity Is Generated

In reference textbooks, the extremely wide diversity of biological entities is traditionally presented as the consequence of genetic changes that occur spontaneously and randomly. However, a growing number of studies in multiple fields now challenge this paradigm of diversity, to the point that it is no longer indefensible. This article attempts to summarize current knowledge about the sources and consequences of genetic and phenotypic diversity in living systems.

As we will see, individual diversity makes ecosystems, species and populations more robust to environmental stress and can favor the emergence of complex collective behaviors such as collectivism. This demonstrates that diversity not only constitutes the fuel for the evolutionary process, but can also confer new functions and better adaptive capacity at all levels of biological complexity. The magnitude of these fitness gains suggests that partial control of the generation of diversity may be beneficial. In keeping with this assumption, numerous examples of tightly regulated mechanisms generating individual genetic and phenotypic diversity have been described among prokaryotes, parasites and complex multicellular organisms. These mechanisms seem to play a key role in the evolution of biological systems as well as in the dynamics of the host–pathogen relationship. Their ubiquity suggests that they are indispensable to adaptation to environmental stress, and that regulated auto-generation of diversity must be considered as a fundamental trait of biological systems. Based on these observations, the present article proposes a new conceptual framework for thinking about the diversity and organization of biological systems. It has three goals in particular: (i) to provide a functional definition of the acquired mechanisms contributing to generate diversity, based on their shared properties, (ii) to identify the factors defining their dynamics, and finally (iii) to discuss the complex question of how and why they were selected and stabilized during evolution.

## The Immeasurable Diversity of Living Entities

We have only recently begun to imagine the true scope of the diversity of life forms and the difficulty that lies ahead to quantify it. Recent estimates of total eukaryotic diversity fall within the range of 1–5 × 10^7^ species (May, 1988; Mora et al., 2011)... with more than 450,000 species of beetles alone, which supposedly led J.B.S. Haldane to say that “*The Creator must have an inordinate fondness for beetles*”... Although only around ten thousand species of prokaryotes have been described, mainly because only a few number of bacteria can be cultivated in the laboratory, indirect molecular approaches based on the annealing of DNA extracted from the environment (without cultivation) suggest that there could be 10^9^ or more prokaryotic species worldwide (Dykhuizen, 1998), and >10^6^ species within a given habitat (Gans et al., 2001). However, even these astronomic numbers do not reflect the real diversity of living forms:

• First, the genotypic diversity within one prokaryotic species can be amazingly high. Species boundaries for prokaryotes appear to be “fuzzy” and members of a bacterial species share certain portions of their genomes (core genome) encoding essential metabolic and informational functions, but often carry unique, strain-specific sequences (auxiliary genes, accessory/flexible/dispensable genome) serving in the adaptation to local environmental pressures (Achtman and Wagner, 2008; Riley and Lizotte-Waniewski, 2009). The size of the core genome appears to vary widely. In the case of *Streptococcus agalactiae*, it accounts for 80% of any single genome of 8 strains (Tettelin et al., 2005) but a recent analysis showed that for *Escherichia coli* it represent only 6% of genes present in 61 strains (Lukjancenko et al., 2010). This fluidity of the prokaryotic genome has led to the emergence of concepts such as the *pan-genome* (core + accessory genome) (Tettelin et al., 2005) and *fuzzy species* (Hanage et al., 2005) in microbiology and each bacterial species is now considered as a “gene flow unit” (Cordero and Polz, 2014).
• Second, the phenotypic diversity of living forms is greater than their genotypic diversity. Prokaryotes (Velicer and Vos, 2009) and protozoa (Li and Purugganan, 2011) may have complex life cycles including multiple differentiation states. Furthermore, multicellular eukaryotic organisms are widely known to display amazing “phenotypic plasticity” (defined as the ability of a single genotype to produce multiple phenotypes in response to variation in the environment) during their development and life. Change can be reversible or irreversible, occur within a single individual or a population, be cyclic or not (reviewed in Piersma and Drent, 2003). Phenotypic plasticity confers the capacity to anticipate predictable seasonal changes or react to unpredictable changes by remodeling physiological processes to compensate for the potentially negative effects of changing conditions. Interest in this phenomenon has surged over the last decades because it could facilitate the accumulation and release of cryptic genetic variation and favor diversification and speciation (Pfennig et al., 2010).
• Third, genetically identical individuals can express considerable variation in behaviors, such as social, sexual, anti-predatory responses, and can show task and diet specialization (reviewed in Dall et al., 2012). While behavioral variation among individuals in eusocial insect societies (queen and various workers) has been described since ancient times, the existence of individual behavioral specialization is now well documented throughout the animal kingdom.

## Biodiversity Increases the Robustness, Evolvability and Collectivism of Biological Systems

“…*there is a constant tendency in the forms that are increasing in number and diverging in character, to supplant and exterminate the less divergent*” Darwin, *The Origin of Species*

The impact of biodiversity on the robustness and productivity of a biological system has been investigated at various levels of complexity.

A growing number of studies have analyzed the consequences of species diversity on the resistance of ecosystems to natural selection. Grassland plant ecosystems have been particularly studied. It has been reported that species-rich communities are generally more productive (Hector, 1999; Tilman et al., 2009), more stable (Tilman and Downing, 1994; Tilman et al., 2006; Proulx et al., 2010) and resistant to extreme climatic changes (Isbell et al., 2015), and are less vulnerable to invasions (Hector et al., 2001) than less diverse system. In controlled laboratory conditions, species diversity also increases the stability of microbial communities against environmental perturbations (Eisenhauer et al., 2012). Likewise, in nature, the reduction of species diversity constitutes a major driver of ecosystem change (Hooper et al., 2012). Multiple mechanisms have been proposed to explain the impact of species diversity on ecosystems, but their relative importance remains controversial (reviewed in Cardinale et al., 2011). Darwin proposed that species diversity might increase the productivity of ecosystems due to the division of labor among species, suggesting that each species is unique in how it exploits its environment (introducing the niche concept). It thus follows that species-rich systems can exploit resources more efficiently than species-poor systems (known as the *complementarity effect*). Alternatively, high species diversity also implies higher chances of having species that efficiently fulfill functions required by their environment (known as the *selection probability effect* or *sampling effect*). A large number of species may imply a certain level of functional redundancy: the loss of one species has a smaller effect in a diverse system than in a species-poor one (known as the *insurance effect*, Yachi and Loreau, 1999). Although more rarely studied (Reusch et al., 2005; Crutsinger et al., 2006; Genung et al., 2010; Cook-Patton et al., 2011; Roger et al., 2012), genotypic diversity within one population of the same species is also thought to improve the resistance of an ecosystem to environmental changes by the same mechanisms.

Studies of microbial populations in controlled environments have clearly demonstrated that even bacteria can display phenotypic heterogeneity independently of genetic or environmental variation, a form of variation that we call “individuality” here. Individuality can result from a random molecular process (*phenotypic noise*), cellular age, asymmetric division, cell-cell interactions or epigenetic modifications (reviewed in Ackermann, 2015) and can have important functional consequences, similar to those of species and genotypic diversity in ecosystems. Individuality can allow certain individuals to survive sudden changes in the environmental conditions [a phenomenon known as *bet hedging* (Philippi and Seger, 1989)]. For example, the persister phenotype promotes *E. coli* survival in the presence of antibiotics (Balaban et al., 2004). Individuality can also lead to the emergence of cheaters (selfish individuals) that exploit the public goods produced by other individuals or favor the cooperative division of labor between individuals (West and Cooper, 2016). Cooperative division of labor enables groups of bacteria to synergize, but also to engage in tasks that are incompatible with each other and thus to gain new functions. For example, multicellular cyanobacteria gain the ability to simultaneously perform photosynthesis and nitrogen fixation though these two tasks are incompatible as the oxygen produced during photosynthesis permanently damages the enzymes involved in nitrogen fixation. Time and space coordination have also been documented, which demonstrates that in some cases bacterial populations cannot be considered as simple assemblies of independent individuals, but must be seen as “functional units.”

In summary, all forms of biodiversity, independently of the level of complexity, seem potentially able to favor the resistance of biological systems to environmental selective pressures. Importantly, this biodiversity can, in many cases, allow for the emergence of new functions within a population and constitute the basis for collectivism. In addition, genotypic and phenotypic diversity may increase the evolvability of populations. Sustained high genotypic diversity could increase the chances of finding and fixing an adaptive genotype. Phenotypic diversity could help maintain genotypic diversity by allowing for the persistence of genotypes in fluctuating environments. Of course, the impacts of diversity are not always positive. High levels of mutation in particular can lead to the accumulation of deleterious mutations that jeopardize the survival of small populations of individuals, though this does not seem to be predominant in nature as a much greater number of positive effects have been reported in the literature.

## Classical Neo-Darwinian Paradigm of Diversity Generation

“*Our ignorance of the laws of variation is profound*” Darwin, *The Origin of Species*

**FIGURE 1:**
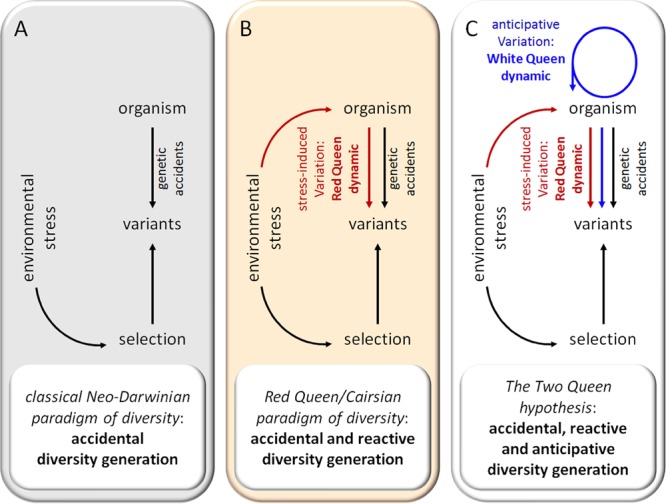
Evolution of paradigms of diversity. **(A)** Classical Neo-Darwinian paradigm. **(B)** Red Queen paradigm. **(C)** The Two Queen hypothesis.

One major goal of evolutionary biology is to understand how living entities diversify. How did a billion species arise from one single species of microbial life 3500 million years ago? Neo-Darwinism hypothesizes that the diversity of living entities is the consequence of genetic changes that occur spontaneously and randomly, without regard for their usefulness, and of the action of natural selection by the environment (**Figure 1A**). In other terms, under this paradigm, genetic accidents are considered as the most important source of diversity and adaptation to environmental stress conditions. However, most spontaneous genetic mutations appear to be neutral. A few are detrimental and beneficial mutations seem to be very rare. This has led to *Muller’s ratchet* concept (Muller, 1964) which postulates that, in absence of genetic mixing, a population accumulates deleterious mutations in an irreversible manner up to a critical threshold inducing its collapse. Three main types of mechanisms to escape this collapse have been described:

• Natural selection has favored the acquisition of mechanisms to control mutation rates and also to ensure some robustness to detrimental mutations. These mechanisms include molecular chaperones, modularity (the separation of networks into clusters, also called the scale-free network organization) and degeneracy (partially interchangeable modules) (Kitano, 2004).
• Antagonistic coevolution acts as a mechanism to purge deleterious mutations from the genome and maintain genotypic diversity among populations (Buckling et al., 2006; Decaestecker et al., 2007; Paterson et al., 2010). Predator and prey, parasite and host and exploiter and victim interact in a continuous “arms race” of defense and counter-defense mechanisms even under constant environmental abiotic conditions. Based on the quote by the Red Queen “*Now here, you see, it takes all the running you can do to keep in the same place*” in Lewis Carroll’s “Through The Looking-Glass” (1871), such dynamics are often called the “**Red Queen”** in evolutionary biology because competitors must constantly evolve to maintain their fitness (Van Valen, 1973). Under the Red Queen dynamic, as summarized by J. B. C. Haldane, “*Just because of its rarity it will be resistant to diseases which attack the majority of its fellows*,” new or rare variants are positively selected.
• In addition, in order to escape the inevitable accumulation of detrimental mutations, organisms have also acquired some mechanisms to open their genome up to genetic innovation and favor phenotypic variation among populations. The nature of these mechanisms appears to differ widely among prokaryotes and multicellular eukaryotes organisms and will be examined below.

## Generation of Genetic Diversity Among Prokaryotes

The sequencing of numerous entire microbial genomes has demonstrated the importance of horizontal gene transfer (HGT) in the evolution of archaeal and bacterial genomes (Gogarten and Townsend, 2005). Bacterial genomes are constantly subjected to the input of new genes from a large available pool. Three mechanisms of HGT that can contribute to the genetic variability and evolution of bacteria have been identified to date (reviewed in Ochman et al., 2000): natural transformation, conjugation, and transduction by phage infection by which exogenous genetic material is presented to a cell as free DNA, as a plasmid or packaged in a phage, respectively. Although each of these processes was characterized decades ago, the availability of numerous complete bacterial genome sequences has sparked renewed interest in their contributions to adaptation and evolution.

HGT-induced genetic mixing may be accidental, but it is generally regulated by both active genetic barriers and environmental stress factors: high population densities, DNA damage, abundance or a lack of certain carbon sources and/or starvation (Galhardo et al., 2007). Genetic barriers include several passive and active mechanisms that ensure genetic stability and relative self/non-self genetic discrimination. Homologous recombinations are strongly favored when the donor DNA and the host genome share DNA sequence similarities required to form DNA heteroduplexes. For example, with a similarity decrease from 100 to 90%, the recombination frequency is reduced by a factor of 40 in *Escherichia coli* (Shen and Huang, 1986). Bacterial restriction-modification (RM) systems are present in over 90% of sequenced bacteria and protect the host DNA against “contamination” by unrelated foreign sequences (Jeltsch, 2003). Diversity-generating retroelement (DGR) systems (Medhekar and Miller, 2007) and CRISP/Cas systems (Brouns et al., 2008) are specific bacterial immune responses against phage infections and regulate transduction-induced HGT. In contrast, several mechanisms may favor HGT. Some bacterial species take up DNA from their close relatives based on short stretches of specific recognition sequences evenly dispersed in the DNA (Smith et al., 1999). Stress-induced activation of SOS-systems provides some help to the DNA integration (Baharoglu et al., 2012). Biofilms, generally defined as “aggregates of microorganisms in which cells are frequently embedded in a self-produced matrix of extracellular polymeric substances that are adherent to each other and/or a surface” (Flemming et al., 2016), have also been reported to be hotspots for HGT (Sørensen et al., 2005). Such negative and positive regulation of genetic exchange delimits “genetic exchange communities” (GECs) constituting a genetic reservoir that includes viruses, bacteria and eukaryotes (Jain et al., 2003; Skippington and Ragan, 2011). GEC boundaries are not necessarily defined by physical proximity or relatedness, but only by the ability to exchange genes by HGT. As rapid adaptation to environmental stress depends on the available genes in GECs, GECs could constitute a natural unit of selection.

In summary, recent advances in microbiology and genetics have led to the conclusion that genetic variation in prokaryotes is not only a “by-product of nature’s imperfections” but is mainly due to HGT mechanisms. It has been estimated (Jain et al., 2003) that HGT could increase the genetic innovation rate 10^7^ to 10^10^-fold in bacteria, suggesting the considerable importance of HGT for bacterial evolution and adaptation. From this perspective, it appear plausible that the genetic code is presently unified mainly because of continuous selective pressures that allow for gene exchange (Syvanen, 1985). As genetic variation due to HGT and SOS systems is regulated and frequently induced in response to environmental stress, these mechanisms appear as reactive diversity generator (DG) mechanisms in prokaryotes. The idea that environmental stress can stimulate the production of genetic variations in prokaryotes (**Figure 1B**) was previously proposed in Cairns et al. (1988) and defended by several authors (Thaler, 1994b). This phenomenon has been variously called “Cairnsian” or “selection-promoted” variation and has been the source of intense polemics due to its quasi-Lamarckian nature. However, almost 30 years later, Cairnsian genetic variations have been largely documented and their molecular mechanisms have now been clarified.

## Diversity Generator Acquisition and Regulation Are Dependent on the Intensity of Environmental Selective Pressures

The relationship between the acquisition of DGs and the intensity of environmental stress is exemplified perfectly by the host–pathogen relationship. To chronically infect a host, bacteria and protozoa must be able to escape the unpredictable and adaptive host immune response, particularly if they persist extracellularly and are permanently exposed to the humoral immune response. Immune escape is frequently made possible by the acquisition during evolution of highly efficient DGs that act constitutively to quickly diversify populations of infectious agents. Whole genome sequencing of bacterial populations during *in vivo* infection has highlighted the within-host evolution ability of various bacterial pathogens (reviewed in Didelot et al., 2016). Bacteria, such as *Pseudomonas aeruginosa*, can rapidly diversify in the host with the presence of hypermutable (mutator) strains that display an increased mutation rate, usually as a result of a loss of functionality of DNA repair systems (Oliver et al., 2000). Many bacterial pathogens, such as *Haemophilus influenzae*, contain genes that are excessively prone to mutation. These hypermutable genes, called “contingency loci,” regulate many aspects of bacterial behavior such as antigenicity, motility, chemotaxis, attachment to host cells, resistance to desiccation, acquisition of nutrients and sensitivity to antibiotics. Bacterial pathogens can also contain “phase-variable” genes that mediate the on/off reversible stochastic switch between variants (reviewed in Moxon et al., 2006). The phase variation is typically associated with external antigens, such as lipopolysaccharide biosynthesis genes and adhesins, iron acquisition genes, as well as type III RM systems (Srikhanta et al., 2005) to thus influence the expression of multiple other genes. The rickettsial pathogen *Anaplasma marginale* has a small genome but uses segmental gene conversion to successively express a great number of variants of the immunodominant Major Surface Protein 2 (Msp2), allowing it to escape from the adaptive immune response (Brayton et al., 2002). Msp2 variants are generated from a small number of donor pseudogenes (<10) with recombination into a single expression site. The number of distinct variants is strongly increased by segmental gene conversion. Expression site mosaics are generated by recombination of short segments from multiple donor alleles. This combinatorial recombination process has the capacity to generate thousands of distinct Msp2 variants and thus allow for lifelong persistence of *Anaplasma* in the host.

Protozoan parasites also possess very specialized diversification mechanisms allowing them to successfully persist in their host. For example, *Trypanosoma brucei* has acquired a mechanism for antigenic variation during infection by which the parasite can turn on and off variant surface glycoprotein (VSG)–encoding genes from a genomic repertoire of ∼2000 different silent genes and pseudogenes mainly located in subtelomeres. This location favors ectopic recombination between VSGs, increasing the diversity of VSGs. Comparison of the VSG expression profile in distinct mice infected with the same *T. brucei* strain demonstrates that each infection presents a distinct sequence of VSGs (Mugnier et al., 2015). In addition, the VSG repertoire appears to be highly diversified between different strains of *T. brucei* (Cross et al., 2014), which suggests that, in nature, each infection may have a private repertoire and sequence of VSGs. *Plasmodium falciparum* erythrocyte membrane protein 1 (PfEMP1) is a diverse family of hypervariable proteins that are inserted into the surface of *P. falciparum*-infected erythrocytes. PfEMP1 mediates binding of infected erythrocytes to the endothelial lining of blood vessels as a strategy to avoid clearance by the spleen and is a major target of host humoral adaptive immunity. PfEMP1 is encoded by a large multi-gene family called *var*. Each parasite genome contains ∼60 *var* genes but many millions of rearranged *var* gene sequences are produced by recombination every 48-h life cycle in infected individuals (Claessens et al., 2014). *Var* genes are expressed in a mutually exclusive manner. The switches in *var* gene expression allow the parasite to evade host immunity and surviving parasites are those that express PfEMP1 variants corresponding to gaps in the endogenous repertoire of host antibodies. Genetic code alterations in the fungal pathogen *Candida albicans* have been shown to also constitute a phenotypic DG (Miranda et al., 2007), creating cell surface variation (Miranda et al., 2013) and modulating drug resistance and immune response (Bezerra et al., 2013).

Diversity generator have been described in helminth parasites such *Schistosoma mansoni*. *S. mansoni* uses the snail as an intermediate host. Specific *S. mansoni* strains can infect only certain snail strains efficiently while others are incompatible. The success of the snail infection is strongly dependent on the expression of polymorphic mucins (SmPoMuc) that form the mucus facilitating parasite penetration through the snail epidermis. Histone modifications of the SmPoMuc promoters generate SmPoMuc transcription polymorphism leading to important phenotypic diversity among the parasite population and favoring success of the infection (Fneich et al., 2016).

In summary, numerous studies have shown that some pathogens, and especially those chronically exposed to the humoral response, have acquired specialized DGs during evolution. These DGs seem to operate constitutively to compensate for the small size, and thereby the poor individual diversity, of the infecting population and to continually escape the host adaptive immune response and persist in the host.

## Multicellular Eukaryotes Acquire Genetic Diversity Generator Mechanisms Acting Anticipatively to Environmental Stress

Frequencies of HGT in unicellular eukaryotes are difficult to quantify, but some reports suggest that HGT between bacteria and protists is not unusual (Andersson, 2005) and is facilitated by the phagocytosis process (Doolittle, 1998). In contrast, multicellular eukaryotes have acquired a specialized tissue layer that provides better protection against environmental conditions and isolates germinal cells from sources of HGT. Therefore, while the impact of HGT on their evolution is not negligible [as illustrated for example by the viral origin of RAG (Kapitonov and Jurka, 2005) and syncitin (Dupressoir et al., 2009) genes in metazoans], HGT frequency in multicellular eukaryotes appears to be dramatically reduced compared to prokaryotes. This phenomenon even affects obligate endosymbiotic bacteria persisting in metazoans that display striking genome stability compared to free-living bacteria (Bordenstein and Reznikoff, 2005). Rare cases of HGT in multicellular organisms appear to be mediated mainly by host infection or the microbiota that constitutes, like an internal biofilm, a hotspot of HGT (reviewed in Skippington and Ragan, 2011). The rarity of HGT in multicellular organisms may be correlated with the acquisition of numerous new DG mechanisms.

Meiosis is one of the major innovations of eukaryotic organisms (reviewed in Cavalier-Smith, 2002; Goodenough and Heitman, 2015; Lenormand et al., 2016). The core genes implicated in the meiosis process appear to be highly conserved among eukaryotes, and this suggests that this mechanism appeared in the last common ancestor of all eukaryotes and has been maintained for over one billion years. The nature of the selective forces that maintain sexual reproduction in most contemporary eukaryotes is a central question of evolutionary biology (reviewed in Lehtonen et al., 2012). In many ways, sexual reproduction is a less efficient method of reproduction compared with asexual reproduction and its costs are high. Mitosis can take as little as 15 min. By contrast, meiosis usually takes more than 10 h. In addition, mates have to be found, special cell types must be formed and diploid genomes need to be maintained.

Functionally, meiosis is a sophisticated form of exchange and mixing of genes between sexual partners. The first step of meiosis, fusion of the gametes, involves specific recognition between sexual partners mediated by multiple highly polymorphic adhesion receptors (Palumbi, 2009). This identity (self/non-self) check is much more restrictive than the genetic barriers governing the bacterial HGT DG process in prokaryotes. It drastically restricts the size and diversity of GEC and sets down sharp boundaries to GEC, which characterizes “classical” metazoan species in opposition to “fuzzy” prokaryotic species. This limited gene mixing could explain why new genes in metazoans arise mainly through the duplication of existing genes (Rubin, 2000). However, the meiosis process also greatly optimizes recombination between sexual partner genomes (Thaler, 1994a), presumably to compensate for poor genetic diversity among partners. Thus, as discussed by Thaler, sexuality appear as a DG mechanism “*enabling creative differentiation within self*” (Thaler, 1994a).

In protists and some lower metazoans, such as Porifera and Cnidaria, reproduction (i.e., an increase in number) is decoupled from sexuality. Like HGT in prokaryotes, sexuality occurs principally in reaction to stressful environmental conditions (Bernstein and Bernstein, 2010). In contrast, in the large majority of higher metazoans, reproduction is closely linked to sexual meiosis. This change is fundamental, as meiosis-induced gene mixing has become an ubiquitous programmed step in an organism’s reproduction. Thus, in higher multicellular organisms, the meiosis DG mechanism initially appeared as a reactive DG in protists, and later became anticipative.

## Multicellular Eukaryotes Acquired Inheritable Phenotypic Diversity Generators

The mechanisms underlying phenotypic variability not associated with evident individual fitness gains have attracted little interest from evolutionary theorists or ecologists. Some of those mechanisms, however, appear to be very interesting when analyzed at the population level.

For example, the fitness gains resulting from development of the adaptive immune system (AIS) during evolution are still the subject of hot debate (reviewed in Muraille, 2014). A large random repertoire of antigenic receptors is costly to develop and could be the source of autoimmune reactions. And yet, despite their drawbacks, AIS-like systems seem to have been independently acquired in several phyla of metazoans, such as jawless vertebrates (Pancer et al., 2004) and arthropods (Watson et al., 2005), with very different anatomies, longevities, and lifestyles. It is well known that the AIS enables an organism to produce a specific immune response to all natural or artificial antigenic structures. For example, the mammal AIS could theoretically generate more than 10^11^, 10^15^ and 10^18^ distinct B cell receptors, αβT-cell receptors and γδT-cell receptors, respectively (Davis and Bjorkman, 1988). However, as the produced repertoire is several orders of magnitude smaller (Arstila et al., 1999), each individual actually generates a unique “private” adaptive immune system (Lu et al., 2014; Thomas et al., 2014). In addition, studies using recent high-dimensional immune system analyses have showed that numerous other parameters of adaptive but also innate immune systems appear to vary widely between individuals, but are remarkably stable over time, thus demonstrating that this variability is not due to an instability or fluctuation of these parameters (reviewed in Brodin and Davis, 2016; Liston et al., 2016). This individual variability has been linked to host genetics, sex, age, microbiota, diet, past/chronic infections and environmental factors. The influence of these factors on the immune system renders the immune response to a pathogenic agent largely unpredictable at the individual level (reviewed in Muraille, 2016). It is frequently neglected that this individualization of immune defenses implies that invasion and escape immune mechanisms developed by pathogens will certainly not always be successful as the specific targets and organization of the immune response are somewhat unpredictable. In a population where individuals display heterogeneous immune responses to infection, the probability that a pathogen is able to infect all individuals could be reduced compared to a homogeneous population. Based on these considerations, I have previously suggested (Muraille, 2014) that the individual diversity of the adaptive immune repertoire is not a by-product of development of the AIS but one of its fundamental properties and could be in part responsible for repeated selection and conservation of the AIS during metazoan evolution. Thus, the generation of a random repertoire of antigenic receptors by the AIS could constitute a neglected ubiquitous anticipative DG in metazoans.

Individual differences in animal behavior that are maintained over time and across contexts constitute the “personality” (Wolf and Weissing, 2012) and have frequently been considered as merely noise by psychologists and ecologists. This paradigm has evolved in light of their roles in the adaptation of a population to stress conditions (Dall et al., 2012). It has become clear that distinct personalities introduce variation in behavior to stress conditions that, at the population level, has led to the emergence of adapted behavior. Recent evidence (reviewed in Singer et al., 2011) suggests that, in mammals, neuronal genomes are genetically diverse and brains are somatic mosaics between individuals. This neuronal genetic diversity results from aneuploidy (whole chromosome gains and losses), genomic copy number variations, and actively “jumping” transposable elements, or “long interspersed repeated sequences” (LINE-1 or L1 elements). New somatic L1 insertions may generate “genomic plasticity” in neurons by causing variation in genomic DNA sequences and altering the transcriptome of individual cells. This random mosaicism of neurons could account for the range of individual differences in behavior observed in isogenic animals presumed to be genetically identical, and could underlie phenotypic discordance in monozygotic twins. It can constitute another example of a widely distributed anticipative DG in metazoans.

Long-term immunological and neuronal memories are widely considered to have been selected because they can facilitate faster adaptation based on the fact that adapted past responses can be also adapted in the future. However, during its lifetime, each individual acquires a unique set of experiences that will influence its future reactions. Thus, long-term memory can also contribute to making an individual’s response to environmental stress singular and unpredictable and can be considered as a so-called “anticipative” DG because the response to a specific stress is not only affected by the previous encounter with this stress, but is also the result of all previous stresses interacting together in a complex non-linear network. In the case of acquired immunity to infectious diseases, it is now clearly established that past infections can deeply affect the immune response to unrelated infectious agents. This phenomenon is partially dependent on the “poly-specificity” of T and B memory lymphocytes and on trained immunity mechanisms (reviewed in Muraille, 2016; Muraille and Goriely, 2017).

It is evident that AIS and the central nervous system are highly complex multitasking systems. Thus, my hypothesis concerning the fitness gains resulting from their generation of individual diversity does not exclude the possible additional contribution of other benefits to their selection during evolution.

## The Two Queen Hypothesis

**FIGURE 2:**
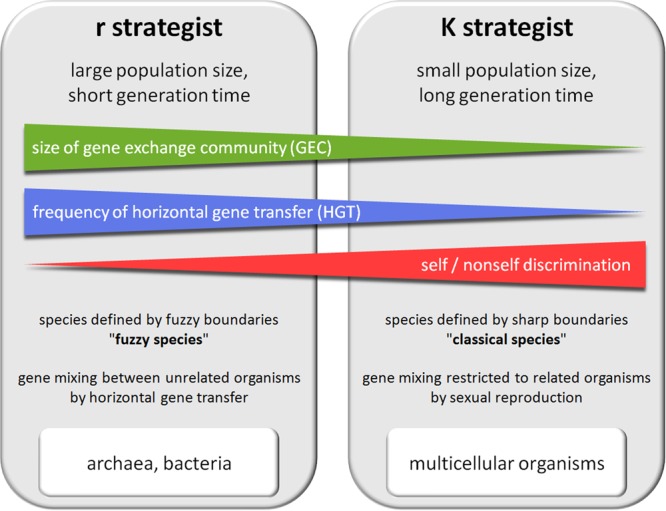
Comparison of prokaryotes and multicellular eukaryotic organisms.

**FIGURE 3:**
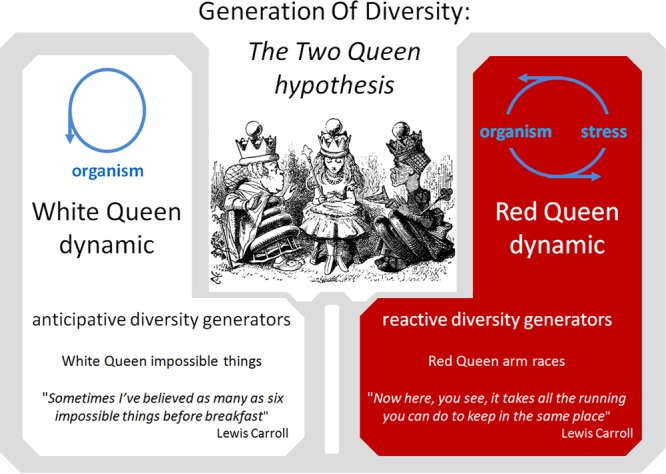
The Two Queen Hypothesis. Comparison of the Red Queen and White Queen diversity generation dynamics.

As previously noted, the activity of some DGs appears to be induced by environmental stress. These reactive DGs participate in the widely described Red Queen/arm race/Cairnsian dynamic. Interestingly, other DG mechanisms, such as DGs acquired by pathogens, seem to act constitutively to allow the pathogen to escape the host adaptive immune response and achieve long-term persistence in the host. Yet other DGs, such as obligatory sexual reproduction, generation of an adaptive immune repertoire and neuronal mosaicism appear to be regulated independently of environmental conditions and can be considered as anticipative DGs. Such anticipative DGs appear mainly in metazoans, suggesting that they are selected in response to particular constraints. In addition to limitations of gene exchange by HGT, most higher metazoans present K selection traits such as a long reproductive life cycle and a small population size (compared to bacteria) (**Figure 2**). These K characteristics drastically reduce the probability that accidental or reactive genetic variations in germinal cells will lead to rapid phenotypic adaptation of metazoan populations. I hypothesize that anticipative DGs have been favored by natural selection in several phyla of higher metazoans because they can overcome these limitations. As a tribute to Leigh Van Valen and Lewis Carroll, I propose to call the dynamic generated by anticipative DGs the “**White Queen**” dynamic, in reference to the White Queen’s famous quote in “Through the Looking-Glass” (1871): “*Sometimes I’ve believed as many as six impossible things before breakfast*”. This metaphor seems particularly appropriate as anticipative DG activities are based on random phenotypic diversification that is not adaptive at individual level (*impossible things*) and anticipative (*before breakfast*) (**Figure 3**).

## Common Characteristics of Diversity Generators

While they may differ in their origin and components, DG mechanisms share common properties:

• Though they are based on deterministic and tightly regulated mechanisms, **DGs confer unpredictability on biological systems**. Two main mechanisms have been described: random generation of genetic diversity (at the germinal or somatic level) and memorization of past experience. For example, in prokaryotes, CRISPR-Cas systems participate in the response to foreign genetic elements such as phages and provide a form of acquired immunity. Their action leads to the integration of new sequences in the prokaryotic genome. Thus, CRISPR-Cas systems diversify prokaryotic populations and customize individuals by integrating part of their immune history at the genome level. In mammals, as previously discussed (Muraille, 2014), it is impossible to predict the repertoire of antigen receptors expressed by an individual under natural conditions. This is due to two distinct DG mechanisms: (i) the random nature of rearrangement of the genes coding for the antigen receptors of lymphocytes, although this process uses a set of enzymes with tightly regulated activity and expression, (ii) the memorization and integration of past immune experience via adaptive immune memory. Accordingly, the adaptive immune responses of wild-type mice in nature display considerable inter-individual differences (Abolins et al., 2017). These two examples show that DGs help make the composition and action of biological systems largely unpredictable, which allows them to partially violate the causality principle, as same causes/conditions do not necessarily lead to similar effects/responses by individuals or populations. Unpredictability itself constitutes a selective advantage during antagonistic coevolution, because the complete adaptation of parasites/predators to unpredictable hosts/prey is impossible, thus confirming the claim of Oscar Wilde that “*One should always be a little improbable.*”
• **DGs operate mainly by manipulating interface control systems** of living entities. HGT and meiosis mechanisms are not limited to producing gene exchange. They define with which partners the genes are exchanged/mixed and thus define GECs (Jain et al., 2003; Skippington and Ragan, 2011). In other words, they determine a form of self/non-self discrimination at the genetic level. Similarly, the AIS regulates self/non-self discrimination that distinguishes between cooperative (microbiota) and selfish (pathogen) microorganisms (Muraille, 2013). The central nervous system of higher metazoans organizes the social interactions and structure of societies. DGs expressed by pathogens specifically target cell surface proteins interacting with the host immune system. Thus, by focusing these effects on the systems that control the interaction of living entities with their environment (termed here “interface control systems”), DGs introduce random variation in response to environmental stress and social interactions and provide for a certain amount of unpredictability at the genetic, phenotypic and behavioral levels. This focus on interface control systems also serves to protect core systems such as metabolic pathways that are highly conserved and interconnected.
• **DGs favor robustness of biological systems against selective pressures mainly at the population level**. As I have previously discussed in the case of the AIS (Muraille, 2014), DGs are generally costly, with unpredictable fitness gains at the individual level, but almost guaranteed fitness gains at the population level. Take the example of eukaryotic meiosis. Phenotypic traits are rarely the consequence of the action of one gene. They are more generally the result of non-linear interactions between the products of a large set of genes (epistasis effect) in a given environment. Thus, by modifying the combination of genes, the meiosis process breaks up successful gene combinations, generating largely unpredictable phenotypes and thereby unpredictable gains at the individual level. The new genotypes in the following generation could be, on average, less fit (Lehtonen et al., 2012). In a Neo-Darwinian paradigm based on the selection of individuals displaying high fitness, this “cost of recombination” makes the fitness gains generated by sexuality difficult to identify. However, these fitness gains become evident at the population level: the resulting phenotypic diversity increases the fitness of a population because a heterogeneous population is more generally resistant to epidemics and more likely to present complementation effects compared to a homogeneous population.

## The Issue of the Selection of Diversity Generators During Evolution

As previously discussed, the action of DGs confers unsure, random fitness gains at the individual level. It thus follows that their expression could be favorable for certain individuals though unfavorable for others. In contrast, at the population level, the fitness gains offered by DGs appear to be more evident and guaranteed. Thus, the issue of the selection of DGs seems to converge with the fundamental problem of the selection and stabilization of cooperative processes between individuals during evolution.

Based on William D. Hamilton’s *inclusive fitness* (or *kin selection*) theory, interacting organisms may have an evolutionary incentive to help each other if they share genes, and the magnitude of this incentive is thought to increase with the degree of relatedness between them. As infection is generally due to a limited number of microbial pathogens, we can reasonably hypothesize that DGs associated with microbial pathogens act within a population composed of closely related individuals. Thus, their selection could be explained from an inclusive fitness selection perspective. However, DGs associated with multicellular eukaryotes, such as meiosis mechanisms and the mechanisms favoring diversity of the adaptive immune response or of the neural network, act clearly within populations composed of unrelated individuals. To explain their selection, we need to use the *multi-level* (or *group selection*) theory, which postulates that natural selection not only acts on individuals but can act (simultaneously) on multiple levels of biological organization, such as cells, individuals, groups or species. Therefore, processes that are disadvantageous at one level can be positively selected for a benefit conferred at a higher level of the biological hierarchy.

Although inclusive fitness and multilevel selection theories are complementary, a long and intense conflict has opposed, and continues to oppose, their partisans (reviewed in Kramer and Meunier, 2016) and, until now, the inclusive fitness theory has dominated the thinking of most zoologists. However, in recent decades, microbiologists have extensively documented the ubiquity of cooperation between populations composed of different species of prokaryotes as well as between viruses, prokaryotes and complex multicellular organisms. The observation that <1% of all known bacteria can be successfully cultured [the “great plate count anomaly” (Staley and Konopka, 1985)] suggests that the majority of bacteria seem to depend on the activity of other bacteria to successfully grow and reproduce (reviewed in Boon et al., 2014). In nature, bacteria often live as biofilms typically containing many unrelated species. This organization has led to the emergence of multiple cooperative mechanisms (complementation, gene exchange, etc.), making biofilms clearly more than the sum of their individual components (Flemming et al., 2016). Accordingly, many bacterial genes appear to only be involved in communication between bacteria and the synchronization of bacterial populations. The ability of bacteria to differentiate in a coordinated manner, such as in the filamentation process (Justice et al., 2008), hyphae formation (Flärdh and Buttner, 2009), differentiation into dormant persister cells (Lewis, 2007), or to exert programmed cell death-like activity (Bayles, 2014) makes more sense if we accept their ability to cooperate and share tasks (West and Cooper, 2016). Multicellular organisms are composed of clonal eukaryotic cells as well as of a great number of bacteria, archaea, fungi, protozoa and viruses, forming together the microbiota that contributes greatly to the fitness of its host. A particularly striking example of this is the symbiosis of termites with an extremely diverse community of microbial gut symbionts. This symbiosis explains the unique ability of termites to degrade lignocellulose, the principal cell wall component of woody plants (Brune, 2014). In mammals, it is now widely accepted that microbiota composition affects the efficacy of the immune system (Rooks and Garrett, 2016), the ability to metabolize nutrients (Rowland et al., 2017) and even mating preference (Sharon et al., 2010) and emotional behavior (Bravo and Forsythe, 2011). On this basis, a new selection unit, the holobiont (“*the sum of the genetic information of the host and its microbiota”* Rosenberg et al., 2009; Rosenberg and Zilber-Rosenberg, 2016), also called the ‘metaorganism’ (“*the macroscopic host and its synergistic interdependence with bacteria, archaea, fungi, and numerous other microbial and eukaryotic species including algal symbionts”* Bosch and McFall-Ngai, 2011), has been proposed. This new level constitutes an important conceptual rupture with previous theories. As written by Rosenberg and Zilber-Rosenberg (2016) “*Animals and plants can no longer be considered individuals*.” By redefining the individual as a consortium of unrelated agents, the holobiont theory postulates that cooperation between unrelated agents is a proven fact. As previously discussed by myself and others (West et al., 2007; Strassmann and Queller, 2010; Muraille, 2013), the problem of the selection and stabilization of cooperative mechanisms in a holobiont/consortium could be solved by the selection of both “identity markers” and “police mechanisms” protecting the consortia of selfish/cheater agents. Thus, the holobiont theory could explain the selection of prokaryotic DGs acting within populations composed of several distant species, such as HGT mechanisms in biofilms in which bacterial immune defenses, such as RM, DGR and CRISPR-Cas systems, are thought to play the role of police mechanisms defining the genetic boundaries of GECs. Of course, the validity of the holobiont theory is not unanimously accepted and several criticisms have been formulated (Douglas and Werren, 2016) regarding in particular the fact that only part of the microbiota is specifically bound to the host.

The repeated selection during evolution of DGs displaying similar properties can be viewed as a neglected example of convergent evolution and suggests that some parts of the evolutionary process are deeply constrained and thus partially predictable. From a cybernetical point of view, a biological system’s need to acquire DGs can be predicted by the *Law of Requisite Variety* of W. Ross Ashby (Ross Ashby, 1956; Ashby, 1958). This law proposed, in substance, that a controller system must contain as much variety (a measure of the number of possible states or actions) as the phenomenon it attempts to control. In our context, this means that biological systems must be at least as diverse that their set of environmental pressures. Thus, under this theory, the survival of biological systems is dependent on their ability to maximize their internal variety (or diversity) to be optimally prepared for any predictable or unpredictable contingency. This principle appears to be true at all levels of complexity as low internal diversity reduced the resistance of an ecosystem (Tilman and Downing, 1994; Hector et al., 2001; Tilman et al., 2006; Proulx et al., 2010; Eisenhauer et al., 2012; Isbell et al., 2015) and species (Reusch et al., 2005; Crutsinger et al., 2006; Genung et al., 2010; Cook-Patton et al., 2011; Roger et al., 2012) to environmental stress. Another fascinating example is the human microbiota. In addition of these metabolic roles, the microbiota forms an integral part of the natural mechanisms of mucosal surfaces that protect the organism against pathogenic agents. A large body of epidemiological data supports the idea that the Western diet (rich in fat and sugar, poor in plant fibers) and lifestyle practices (Caesarian sections, antibiotic use, and formula feeding of infants) have led to a dramatic loss of diversity of the human gut microbiota in developed countries compared to the human population living in developing countries and non-human primates (Moeller et al., 2014; Martínez et al., 2015; Clayton et al., 2016). This loss of diversity appears to be strongly correlated with the development of immune pathologies such as inflammatory bowel disease, autoimmune diseases (i.e., multiple sclerosis, type 1 diabetes, and rheumatoid arthritis), obesity-associated metabolic disorders and allergies (reviewed in Silva et al., 2015), suggesting that individual internal microbial diversity is required to educate the immune system and allow it to adequately respond to environmental stress.

In conclusion, the selection of DGs constitutes a fascinating and complex problem that challenges the theories explaining the selection of cooperative processes during evolution. As the majority of DGs are unrelated multitasking systems, there is no reason why all DGs would have been selected in the same way or on the basis of a single property. I remind the reader that many complex mechanisms have been described and analyzed over decades, though no consensus regarding the reasons for their selection has emerged. Thus, this article does not aim to choose between the different theories explaining selection of the DGs, but only to document and provide a formal definition of the DG mechanism and analyze the consequences of the actions of DGs.

## Overall Conclusion

The theory of evolution must not be dogmatic and must evolve as our knowledge grows. The “modern synthesis,” also called Neo-Darwinism, forged in the 1930s and 1940s, has combined genetic and natural selection. Since then, generations of evolutionary biologists have extended the framework of Neo-Darwinism to include multilevel selection, the interdependent cooperative nature of genomes, extra-genetic inheritance, niche construction, the developmental process and phenotypic plasticity (reviewed in Corning, 2008; Rosenberg et al., 2009; Fusco and Minelli, 2010; Moczek et al., 2011; Laland et al., 2014, 2016). It is now time to integrate new knowledge about the mechanisms generating genetic and phenotypic diversity. Numerous authors have already brilliantly discussed the importance of stress-induced genetic diversity in unicellular prokaryotes and eukaryotes (reviewed in Thaler, 1994b) and its ability to generate a Red Queen dynamic. Here, I have tried to summarize the observations justifying introduction of the concept of the DG mechanism. Formally, I propose to define DGs as ***mechanisms that generate unpredictability of the composition and/or behavior of biological systems by acting on their interface control systems, which leads to random alterations in their response to environmental stress***. As a growing body of data strongly suggests that biodiversity increases survival to environmental stress at all levels (ecosystem, species, population and individual) of biological complexity, it is not really surprising that evolution processes have favored the selection of specific mechanisms to regulate the emergence of diversity among populations. Interestingly, the existence of DGs profoundly changes the dynamics of evolution. In contrast to the classical Neo-Darwinian paradigm where diversity is generated passively by accidental events, living entities also appear able to actively generate diversity in reaction to stress (Red Queen/Cairnsian dynamic) and/or anticipatively at certain points in time (White Queen dynamic) (**Figure 1C**). As discussed previously, the predominance of the Red Queen and White Queen dynamics seems to depend on the r/K selection traits of organisms. Under this new vision, evolution is conceived as a more “intelligent” and Lamarckian-like process than is generally accepted. Though genetic diversity due to DGs remains random, it is regulated in time and space. DG activities are generally focalized on particular sets of genes over a short period of time, as observed with phase variation in bacteria (Moxon et al., 2006), the meiosis recombination process [hot spot of recombination (Paigen and Petkov, 2010)] and the AIS [hypermutation of immunoglobulin genes (Martin and Scharff, 2002)].

Diversity generators play a key role in the host/pathogen relationship. Both the host and the pathogen display DG mechanisms and a large part of their interactions are dependent on these mechanisms. The host/pathogen relationship can, in part, be seen as a competition between DGs at the population level. The importance of individual diversity in the control of epidemics is currently not taken into account in vaccine strategies. In the context of vaccination campaigns, it might be important to avoid uniformization of the immune responses. Thus, as I suggested previously (Muraille and Goriely, 2017), it could be of interest to administer distinct vaccines targeting the same pathogen within a given population.

Diversity generator play also a key role in the evolution of living entities. DGs could favor diversification and speciation in two distinct ways: (i) By acting on the genome of germinal cells, some DGs, such as HGT and meiosis mechanisms, directly affect diversification and speciation by producing new alleles. If the effects of DGs are not inheritable, as in the case of phenotypic diversity generated by the AIS and neuron mosaicism that are limited to a fraction of somatic cells and thus do not affect germinal cell lines, DGs can favor genetic robustness and thus preserve rare alleles and help maintain genetic diversity in populations that also favor the speciation process. (ii) DG activities can also lead to an increasing complexity of social organization inside biological systems. It is therefore likely that they participated in the emergence of complex systems such as eukaryotes, multicellular organisms and societies. Each of these systems can be viewed as a consortium of cooperative agents (Muraille, 2013). By increasing the individuality of the agents in a system, DGs favor the possibility of complementation between these agents and thereby their interdependence and thus the global complexity of the system. Thus, DGs appear to be essential elements promoting growth of complexity during evolution.

Seeing the generation of diversity as the consequence of the action of regulated DGs leads us to two important general conclusions about life properties and the evolution process: (i) Biodiversity should no longer be seen only as a “characteristic of life” and the result of accidental processes but as indispensable to the survival of life and actively generated by the latter. Thus, **auto-generation of diversity can be viewed as a fundamental property of biological systems**. From this perspective, the actual lack of biodiversity observed in a great number of ecosystems (Miraldo et al., 2016) should be alarming as our proper survival is dependent on our ecosystems and their robustness is dependent on their biodiversity. (ii) Genetic diversity is traditionally viewed as the fuel of the evolutionary process. As both DGs acting on the genome (HGT and meiosis mechanisms) require partners, we must conclude that **adaptation to environmental conditions, and more generally the evolution of biological systems, can be viewed as the result of a social cooperative process** where genes can be considered as “public products” [the public goods hypothesis (McInerney et al., 2011)] shared in a GEC. This vision is diametrically opposed to the classical Neo-Darwinian evolution paradigm where diversity remains mainly due to accidental alteration of the genome of individuals living only in competition with others.

Finally, from a theoretical point of view, diversity, self-organization (i.e., the ability to spontaneously organize without external tuning) and unpredictability have always been considered as characteristics of biological systems that are fundamental though difficult to reconcile (Coffey, 1998). Recognizing the existence of regulated DGs could allow for a better understanding of how biological systems can diversify and display both ordered and unpredictable patterns.

## Theoretical Predictions

A theoretical scientific framework must satisfy Popper’s refutability criteria by presenting testable predictions. In this article, I have tried to synthesize current knowledge about the mechanisms of diversity generation. In opposition to the classical Neo-Darwinian paradigm presenting diversity exclusively as the result of accidental processes, I propose that a large part of genetic and phenotypic diversity is the consequence of regulated mechanisms that I call Diversity Generators (DGs). I propose two experimentally refutable predictions about DGs:

• **DGs are indispensable to the survival of living entities, and therefore all natural biological entities must have at least one mechanism that meets the definition of a DG.** To my knowledge, HGT mechanisms have been described in all bacteria and Archaea and the large majority of eukaryotic organisms display meiotic/sexual reproduction. The rare metazoans, like Rotifera, that have lost sexual meiosis and remain diversified seem to present high levels of HGT (Flot et al., 2013; Debortoli et al., 2016), which could compensate for the absence of sexual gene mixing.

• **The dynamics of DGs depend on the intensity of environmental selective pressures and of the r/K strategy of organisms.** As previously discussed and summarized in **Table 1**, this prediction seems largely validated by (i) the acquisition of specialized constitutive DGs by pathogens permanently exposed to a host adaptive immune response, and (ii) the acquisition of anticipative DGs by complex multicellular organisms to compensate for their K selection traits, such as a long reproductive life cycle and a small population size.

**Table 1:** Summary of the association between diversity generation dynamics and r/K selection strategies.

	Population size	Generation time	Selective pressure	DG mechanisms	DG dynamic
**Bacteria, Archaea**	Large	Short	Fluctuating	HGT-mediated genetic mixing	**Reactive**
**Examples of pathogenic bacteria:**					
*Pseudomonas aeruginosa*	Small	Short	Strong and constant	Hypermutable strain	Constitutive
*Haemophilus influenzae*	Small	Short	Strong and constant	Contingency locus, phase variation	Constitutive
*Anaplasma marginale*	Small	Short	Strong and constant	Segmental gene conversion	Constitutive
**Protists and lower Metazoa**	Large	Short	Fluctuating	Meiosis-mediated genetic mixing	**Reactive**
**Examples of parasites:**					
*Trypanosoma brucei*	Small	Short	Strong and constant	VSGs	Constitutive
*Plasmodium falciparum*	Small	Short	Strong and constant	PfEMP1	Constitutive
*Candida albicans*	Small	Short	Strong and constant	Genetic code alterations	Constitutive
*Schistosoma mansoni*	Small	Short	Strong and constant	SmPoMuc	Constitutive
**Higher Metazoa**	Small	Long	Fluctuating	Meiosis-mediated genetic mixing, Adaptive Immune System, Central Nervous System	**Anticipative**

## Societal Implications

The theory of evolution constitutes the conceptual foundation of modern biology and consequently of the life sciences. Unfortunately, its impact on the organization of human societies remains negligible. For example, education and fundamental research are subject to an increasing number of evaluation criteria. Although these controls were initially developed to optimize education and fundamental research, they also serve to standardize them. Is it reasonable to homogenize the intellectual formation of individuals and research activities, while diversity is a source of robustness, synergy and complexity in all living systems? More worryingly, global population growth will require sustained food production during the 21st century. However, the industrialization of agriculture over the past 50 years has led to a dramatic fall in the diversity of agricultural products. Plants and animals have been intensively selected for strength and productivity. While this strategy led to good results over the short term, it is reasonable to doubt the ability of standardized populations to resist future climate changes that will likely lead to the emergence of new pathogens. Must I remind the reader that a particular genotype/phenotype is optimally adapted only to a given set of environmental conditions? There are no phenotypes that perform perfectly in all situations. Thus, there is an urgent need to reconsider the importance of diversity within populations, both from a theoretical and a practical point of view.

## Author Contributions

The author confirms being the sole contributor of this work and approved it for publication.

## Conflict of Interest Statement

The author declares that the research was conducted in the absence of any commercial or financial relationships that could be construed as a potential conflict of interest.
